# Differences in Prefrontal, Limbic, and White Matter Lesion Volumes According to Cognitive Status in Elderly Patients with First-Onset Subsyndromal Depression

**DOI:** 10.1371/journal.pone.0087747

**Published:** 2014-01-31

**Authors:** Jun-Young Lee, Soowon Park, Scott Mackin, Michael Ewers, Helena Chui, William Jagust, Philip S. Insel, Michael W. Weiner

**Affiliations:** 1 Department of Psychiatry and Behavioral Science, Seoul National University College of Medicine, SMG-SNU Boramae Medical Center, Seoul, Republic of Korea; 2 Center for Imaging of Neurodegenerative Diseases, Veterans Affairs Medical Center, San Francisco, California, United States of America; 3 Department of Psychiatry, University of California San Francisco, San Francisco, California, United States of America; 4 Institute for Stroke and Dementia Research, Ludwig Maximilian University, München, Germany; 5 Department of Neurology, University of Southern California, Los Angeles, California, United States of America; 6 School of Public Health and Helen Wills Neuroscience Institute, University of California, Berkeley, California, United States of America; 7 Department of Radiology, Psychiatry, Neurology, and Medicine, University of California San Francisco, San Francisco, California, United States of America; Nathan Kline Institute and New York University School of Medicine, United States of America

## Abstract

The purpose of this preliminary study was to test the hypothesis that subsyndromal depression is associated with the volume of medial prefrontal regional gray matter and that of white matter lesions (WMLs) in the brains of cognitively normal older people. We also explored the relationships between subsyndromal depression and medial prefrontal regional gray matter volume, limbic regional gray matter volume, and lobar WMLs in the brains of patients with mild cognitive impairment (MCI) and Alzheimer's disease (AD). We performed a cross-sectional study comparing patients with subsyndromal depression and nondepressed controls with normal cognition (*n* = 59), MCI (*n* = 27), and AD (*n* = 27), adjusting for sex, age, years of education, and results of the Mini-Mental State Examination. Frontal WML volume was greater, and right medial orbitofrontal cortical volume was smaller in cognitively normal participants with subsyndromal depression than in those without subsyndromal depression. No volume differences were observed in medial prefrontal, limbic, or WML volumes according to the presence of subsyndromal depression in cognitively impaired patients. The absence of these changes in patients with MCI and AD suggests that brain changes associated with AD pathology may override the changes associated with subsyndromal depression.

## Introduction

Depression is a leading cause of disability worldwide. Late-life depression causes more disability and a higher number of suicide attempts than does depression in young adults [1]. A diagnosis of major depression using the Diagnostic and Statistical Manual of Mental Disorders, Fourth Edition (DSM-IV) is made when the following are present: (1) either depressed mood or loss of interest, (2) five or more depressive symptoms co-occurring and persisting over a 2-week period, and (3) functional impairment [2]. Subsyndromal depression has been usually defined as the presence of 2 or more depressive symptoms over a 2-week period and functional impairment and it does not meet the crieteria of major depressive disorder and dysthymia [3], [4]. The term of subsyndromal depression is generally used for milder form of depression than major depressive disorder and risk of it [5].

Clinical studies indicate distinct etiological factors and symptoms in late-life depression compared with depression earlier in life [6]. Subsyndromal depression is the more common form of late-life depression, with a prevalence rate three times higher than that for late-life major depression (point prevalence, 13–30%) [7]. Subsyndromal depression is associated with serious functional impairment, and 25% of patients with subthreshold depression develop major depression within 2 years [8]. In addition, people, who have subsyndromal depressive symptoms, showed 5.5 times higher risk developing first onset major depression in the next year than without the depressive symptoms [9]. Therefore, it is important to identify neuroanatomical changes associated with late-life subsyndromal depressive disorder to understand its initiating mechanisms.

Late-life major depression has been consistently linked to brain abnormalities. Many neuroimaging studies have found that late-life major depression is associated with decreased medial frontal gray matter volume [10], [11] and an increased number of white matter lesions (WMLs) [12], [13], but the relationship between late-life major depression and limbic system abnormalities remains unclear [14]–[16]. Late-life subsyndromal depression could be related to these brain structures if it shares common neuroanatomical substrates with late-life major depression. However, only a few studies have reported the associations between late-life subsyndromal depression and low frontal volumes [17], and the relationship between late-life subsyndromal depression and WMLs and the limbic system has not been reported. Therefore, we hypothesized that the volume of the medial prefrontal cortex and that of WML are larger in patients with late-life subsyndromal depression than in nondepressed controls among cognitively normal elders.

Furthermore, subsyndromal depression is more common in patients with mild cognitive impairment (MCI) and Alzheimer's disease (AD) than in their healthy counterparts [18], and depression is also thought to be prodromal to or a risk factor for cognitive impairment [19]. Some studies have reported that [20], [21] medial temporal atrophy and Alzheimer's pathology are not associated with depressive symptoms in patients with AD, suggesting that regional brain changes are not always necessary for depressive symptoms to appear. However, no study has explored the relationships between regional brain changes and subsyndromal depression in cognitively impaired elders. Therefore, in this study, we explored the relationships among medial prefrontal regional gray matter volume, limbic regional gray matter volume, and lobar WMLs in patients with MCI or AD who had subsyndromal depression and tested whether cognitive impairment was predicted by these regional brain changes or by subsyndromal depression itself.

## Methods

### Subjects and evaluation

In total, 113 subjects were included: 59 normal controls (NC), 27 patients with MCI, and 27 patients with AD. Subjects were a convenience sample recruited from three academic dementia centers (University of California Davis, University of California San Francisco, and University of Southern California), between 2007 and 2010 as part of a multicenter collaborative study examining the effects of cerebral vascular disease and AD on cognitive functioning and brain structure changes.

Exclusion criteria were age <55 years, non-English speaking, severe dementia (Clinical Dementia Rating [CDR] >2) [22], evidence of alcohol or substance abuse, head trauma with loss of consciousness lasting >15 minutes, severe medical illness, neurologic or psychiatric disorders other than dementia, or currently taking medications likely to affect cognitive functioning [23]. Additionally, subjects were excluded if brain magnetic resonance imaging (MRI) showed evidence of cortical infarction, hemorrhage, or structural brain disease other than atrophy, lacunae, or WMLs [24]. All participants underwent a complete medical history, assessment of activities of daily living, physical examination, neurologic examination, neuropsychological testing, serum chemistry, blood counts, vitamin B12 level assessment, syphilis serology, thyroid function tests, and a structural MRI examination.

Participants were grouped as D+ or D− by the presence or absence of subsyndromal depression. Trained psychiatrists diagnosed subsyndromal depression according to the DSM-IV using the Structured Clinical Interview, which requires that the patient have two or more simultaneous symptoms listed in the DSM-IV depressive episode criteria [2]. These symptoms must have been present most or all of the time for a period of at least 2 weeks and must have been associated with evidence of functional dysfunction. Additionally, the individual must not have met the criteria for diagnosis of major depression or dysthymia. The presence of either low mood or loss of interest or pleasure was not considered because no clinical differences have been observed between subjects with depression with and without these symptoms [4]. We interviewed all participants and caregiver informants using the Minimum Uniform Data Set [25]. Information on prior episodes of depression and antidepressant medication history were also obtained. We excluded patients who met the criteria for major depressive disorder, had a previous depressive episode, or were taking antidepressant medication. Therefore, all patients with subsyndromal depression reported the present episode as their first depressive episode.

Dementia was diagnosed using the National Institute of Neurologic and Communicative Disorders and Stroke and the Alzheimer's Disease and Related Disorders Association [26] diagnostic criteria for AD. Participants were grouped by their levels of cognitive impairment into NC (CDR score = 0), MCI (CDR = 0.5), or AD (CDR≥1) groups. We excluded patients with subcortical ischemic vascular dementia to make the dementia group homogeneous.

All participants were administered the Mini-Mental State Examination (MMSE) [27] and a standardized battery of neuropsychological tests. The memory score was calculated based on the Word List Learning tasks of the Memory Assessment Scale [28] using methods based on item-response theory and were then transformed to a standard scale with a mean of 100 and a standard deviation of 15 [29].

### Ethics Statement

This study was reviewed and approved by the institutional review boards of all study sites (University of California Davis, University of California San Francisco, and University of Southern California), and written informed consent was obtained from the study participants or their legal representatives.

### MRI data acquisition and processing

The entire brain was imaged using a 1.5T Magnetom VISION system (Siemens; Erlangen, Germany) equipped with a standard quadrature head coil. We derived WML volume from proton density-weighted and T2-weighted spin-echo axial images from a double spin-echo sequence (repetition time [TR]/echo time [TE]1/TE2, 2500/20/80 msec; in-plane resolution, 1.0×1.4 mm^2^; slice thickness, 3 mm oriented along the anterior–posterior commissure line) and regional frontal gray matter volumes from T1-weighted coronal three-dimensional images from the magnetization-prepared rapid gradient echo (TR/TE/TO = 13.5/7/300 msec; in-plane resolution, 1.0×1.0 mm^2^; slice thickness, 1.4 mm oriented along the long axis of the hippocampus).

Total WML volume and total intracranial volume (TIV) were based on the multichannel segmentation of expectation maximization segmentation [30], and lobar WML segmentation was based on atlas-based deformable registration method [31]. The technical details of these procedures have been described previously [32].

Frontal regional cortical and limbic system volumes were determined using Freesurfer image-analysis software ver. 4.0 (http://surfer.nmr.mgh.harvard.edu/). The technical details of those procedures have been described previously [33]. Cortical volume combined both thickness and area information, and subcortical volume was based on voxel numbers. Parcellation of the cerebral cortex into units was based on gyral and sulcal structures [34].

### Statistical analysis

Missing data were excluded from the analyses. Pearson's correlation analysis was used to find the basic relationships among medial prefrontal regional gray matter volume, limbic regional gray matter volume, and lobar WML.

We used Student's *t*-test to compare subjects' clinical characteristics and analysis of covariance to compare the regional gray matter volumes of the anterior cingulate cortex, medial orbitofrontal cortex, hippocampus, and amygdala and the WML volumes of frontal, parietal, temporal, and occipital regions between the D− and D+ groups after adjusting for sex, age, years of education, and the MMSE score to control for the impact of demographic and cognitive effects on brain atrophy and depression in each cognitive group. The Benjamin and Hochberg method was used to correct for multiple comparisons. A two-tailed *p*<0.05 was considered significant.

We used multiple linear regression models to predict cognition. Independent variables were regional brain volumes, the presence of subsyndromal depression, age, gender, and years of education. The dependent variables were MMSE and memory scores. Regression model assumptions were examined by residual plots before data analysis.

All regional brain volumes and WML volumes were normalized to each TIV according variations in head size: VOLn = VOLr×TIVm/TIVr, where VOLn and VOLr are the normalized volume and the raw volume of a subject, respectively, and TIVm and TIVr are the mean TIV from all subjects and the TIV of the subject, respectively. TIV was calculated by adding the volume of all voxels classified as cerebrospinal fluid or brain tissue from frontal, temporal, parietal, occipital, and subcortical areas, excluding voxels classified as the brain stem and cerebellum. The volume unit was cm^3^. WML volumes were log-transformed to improve model fits.

## Results

Table 1 shows the demographic and clinical data for the 113 subjects according to the presence of subsyndromal depression. D+ group showed larger frontal WML volume and smaller right medial orbitofrontal cortical volume than D− group in NC participants (Table 2). Frontal WML volume and right medial orbitofrontal cortical volume were highly correlated (*r* = −0.47, *p*<0.001). Right amygdala volume tended to be larger in patients in the D+ group with MCI than that in the D− group (mean difference, 0.2; standard error [SE], 0.05; unadjusted *p* = 0.02), but this difference was not significant after adjusting for multiple comparisons (corrected *p* = 0.09). No differences were observed between the D+ and D− groups of patients with AD in medial prefrontal, limbic, or lobar WML volumes.

**Table 1:** Demographic and clinical characteristics of all participants (n = 113).

	NC		MCI		AD	
	Control (*n* = 52)	Depression (*n* = 7)	Control (n = 18)	Depression (*n* = 9)	Control (*n* = 15)	Depression (*n* = 12)
Female, n	30 (58%)	1 (14%)	5 (28%)	3 (33%)	12 (80%)	5 (42%)
Age, y	72.9 (7.1)	80.9 (6.3)	73.5 (7.1)	70.1 (6.5)	78.7 (7.0)	76.8 (8.7)
Years of education	15.5 (3.3)	16.1 (2.7)	15.5 (3.0)	16.1 (2.5)	13.3 (3.3)	13.8 (2.0)
MMSE	29.2 (1.1)	29.3 (0.5)	27.7 (1.6)	28.1 (1.9)	22.7 (4.1)	21.8 (4.6)
Memory score	108.9 (15.5)	95.1 (12.3)	84.6 (10.6)	94.8 (16.4)	61.4 (15.6)	56.6 (12.5)
CDR	0 (0.1)	0 (0)	0.5 (0.2)	0.6 (0.2)	0.9 (0.5)	1.1 (0.6)

Mean (standard deviation). MMSE, Mini-Mental State Examination; CDR, clinical dementia rating; NC, normal control; MCI, mild cognitive impairment; AD, Alzheimer's disease.

**Table 2:** Mean volumes of lobar white matter lesions (WMLs), regional medial prefrontal cortices, and limbic structures according to the presence of subsyndromal depression and cognitive status.

Brain area	NC			MCI			AD		
	D− (*n* = 52)	D+ (*n* = 7)	*p*-value	D− (*n* = 18)	D+ (*n* = 9)	*p*- value	D− (*n* = 15)	D+ (*n* = 12)	*P*-value
WML									
Frontal WML	7.0 (7.4)	17.1 (7.0)	0.01	9.2 (6.0)	8.9 (3.4)	0.56	14.0 (14.6)	15.5 (14.4)	0.44
Parietal WML	2.4 (2.8)	5.5 (3.3)	0.24	3.6 (3.0)	2.2 (1.1)	0.88	3.8 (5.8)	4.0 (4.2)	0.44
Temporal WML	2.9 (1.1)	3.7 (1.8)	0.37	2.7 (0.8)	2.9 (0.9)	0.68	2.9 (2.0)	2.9 (2.1)	0.44
Occipital WML	0.9 (0.6)	1.5 (1.8)	0.70	1.1 (0.5)	0.9 (0.4)	0.68	1.4 (1.4)	1.1 (0.7)	0.44
Prefrontal cortex									
Left ACC	4.1 (0.5)	3.8 (0.6)	0.14	3.8 (0.7)	4.1 (0.5)	0.66	4.1 (0.7)	3.8 (0.8)	0.68
Right ACC	3.9 (0.6)	4.0 (0.5)	0.67	3.7 (0.8)	3.7 (0.8)	0.66	3.5 (0.6)	3.3 (0.8)	0.68
Left medial OFC	4.1 (0.5)	3.8 (0.7)	0.88	3.6 (0.5)	4.2 (0.6)	0.18	3.6 (0.6)	3.6 (0.5)	0.68
Right medial OFC	4.4 (0.5)	3.5 (0.6)	0.03	4.0 (0.6)	4.3 (0.4)	0.66	3.9 (0.5)	3.9 (0.7)	0.68
Limbic system									
Left hippocampus	3.9 (0.5)	3.4 (0.3)	0.76	3.4 (0.6)	3.7 (0.5)	0.64	2.8 (0.6)	2.7 (0.4)	0.72
Right hippocampus	3.9 (0.5)	3.6 (0.3)	0.76	3.4 (0.6)	3.7 (0.5)	0.74	2.9 (0.7)	2.7 (0.3)	0.72
Left amygdala	1.5 (0.2)	1.3 (0.2)	0.76	1.3 (0.2)	1.5 (0.1)	0.30	1.1 (0.3)	1.0 (0.2)	0.72
Right amygdala	1.6 (0.5)	1.5 (0.6)	0.76	1.4 (0.2)	1.6 (0.2)	0.09	1.2 (0.3)	1.2 (0.1)	0.72

Analysis of covariance with the Benjamin and Hochberg's correction was used to adjust for sex, age, years of education, and the Mini-Mental State Examination (MMSE) score. Mean (standard deviation). All WML volumes were normalized to total intracranial volume. WML volumes were log-transformed. Age, gender, years of education, and the MMSE score were adjusted before comparison. *P*-values were adjusted for multiple comparisons. ACC, anterior cingulate cortical volume; OFC, orbitofrontal cortex; NC, normal control; MCI, mild cognitive impairment; AD, Alzheimer's disease; D−, without subsyndromal depression; D+, with subsyndromal depression.

**Figure 1:**
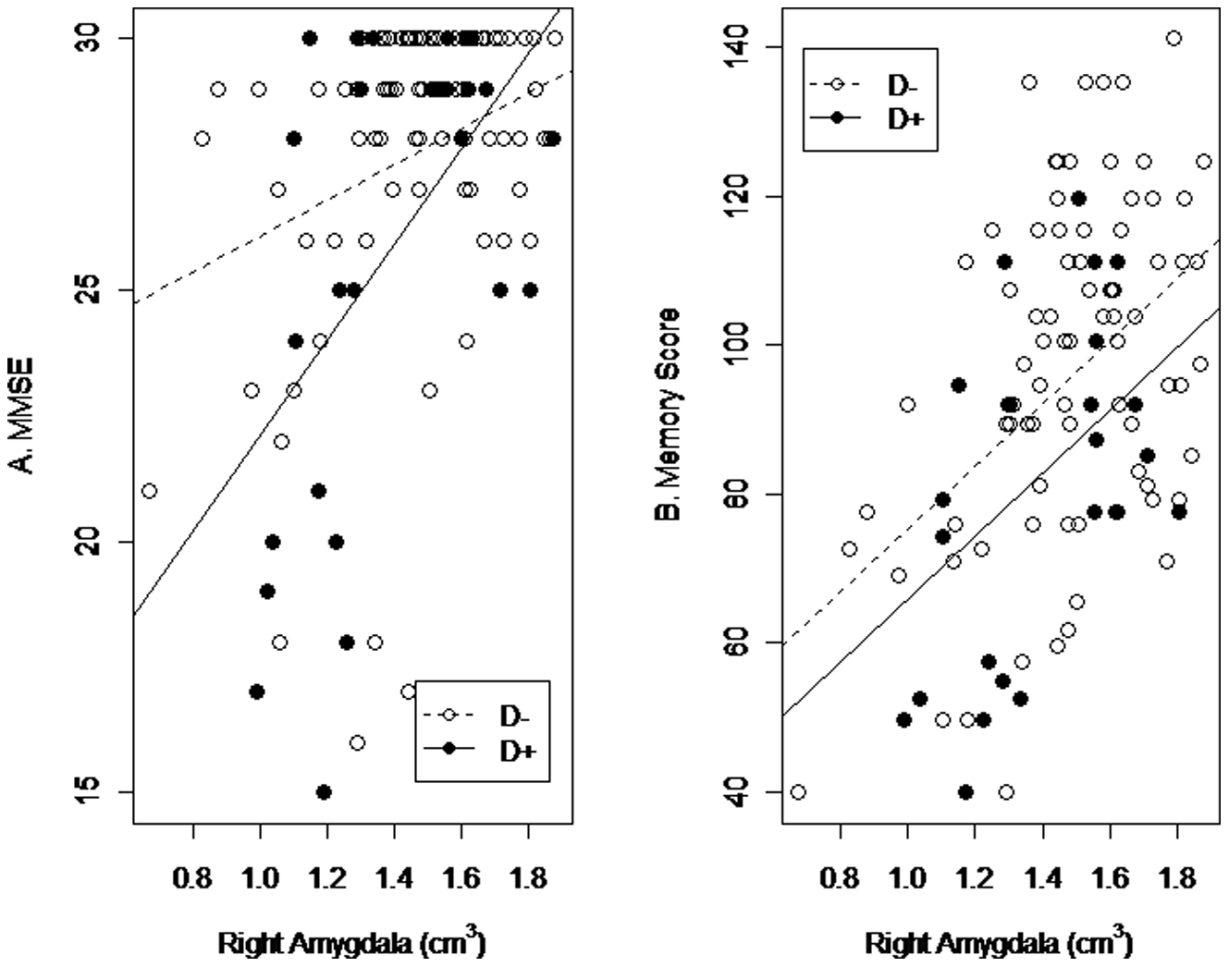
Relationship of right amygdala volume with A. Mini-Mental State Examination (MMSE) score (β, 3.6; standard error [SE], 1.4, *p* = 0.01) and B. memory score (β, 42.0; SE, 8.5; *p*<0.001) across all cognitive groups. Covariates are right medial orbitofrontal cortex, age, gender, years of education, and the presence of subsyndromal depression. The presence of subsyndromal depression lowered the MMSE intercept (β, −9.9; SE, 3.7; *p* = 0.01) and memory score significantly (β, −9.4; SE, 4.7; *p* = 0.05). D−, without subsyndromal depression; D+, with subsyndromal depression.

We conducted multiple linear regression analysis to assess the relationships of brain regions (frontal WML, right medial orbitofrontal cortical volume, right amygdala) that showed significant differences in prior analyses with subsyndromal depression and cognition across the D+ and D− diagnostic groups after controlling for age, gender, and years of education. The MMSE score was predicted by right amygdala volume (β, 3.6; SE, 1.4; *p* = 0.01) (Figure 1A). The presence of subsyndromal depressive symptoms lowered the intercept significantly (intercept change, −9.9; SE, 3.7; *p* = 0.01) and increased the slope of this association (subsyndromal depression×right amygdala volume interaction: slope change, 6.0; SE, 2.6; *p* = 0.02). Memory score was also predicted by right amygdala volume (β, 42.0; SE, 8.5; *p*<0.001) (Figure 1B). The presence of subsyndromal depression lowered the intercept significantly (intercept change, −9.4; SE, 4.7; *p* = 0.05) and was not associated with the interaction between memory and amygdala volume (subsyndromal depression×right amygdala volume interaction: slope change, 9.8; SE, 19.8; *p* = 0.62). These results show that the presence of subsyndromal depression predicted cognitive function independent of right amygdala volume. Frontal WML and right medial orbitofrontal cortical volume were not associated with the MMSE or memory scores.

## Discussion

This is the first study to explore the relationships between subsyndromal depression and structural changes in cognitively normal, MCI, and AD groups. The major findings of this study are as follows: 1. Subsyndromal depression was related to frontal WML and right medial orbitofrontal cortical volume in cognitively normal elders, which was similar to previous findings in patients with late-life major depression. 2. In contrast, subsyndromal depression was not related to medial prefrontal, limbic, or WML volumes in patients with MCI or AD, although patients with MCI and subsyndromal depression showed a tendency toward a larger volume in the amygdala compared with that of patients with MCI without subsyndromal depression. These findings suggest that the effects of AD pathology may override the effects of depression on brain structure in patients with MCI or AD. Each of these findings will be discussed below.

We found that the right medial orbitofrontal cortical volume was smaller in cognitively normal elderly patients with subsyndromal depressive disorder than in non-depressed participants. Kronhaus et al. [35] found the decreased ventral prefrontal activity in subsyndromal depressive patients. Kumar et al. [36] also reported that late-onset subsyndromal depression was related to low prefrontal volumes similar to late-onset major depression. We also found that frontal WML volume was greater in the group with subsyndromal depression and was associated with orbitofrontal atrophy. Previous studies of elderly adults found that the prevalence of depressive symptoms increased as the volume of frontal WML [32] or subcortical WML [37] increased. However, no published studies have investigated WML in elders with subsyndromal depression. WMLs are common in healthy elders, reflecting cerebrovascular pathology resulting from ischemia, arteriosclerosis, and incomplete infarction [38]. Because only frontal WMLs are related to subsyndromal depression, this finding supports the vascular depression hypothesis, namely that disruption of the frontostriatal circuit by WML predispose depression [39]. These findings are concordant with other late-life major depression studies [12], [36] and show that subsyndromal depression in older adults has common neuroanatomical abnormalities comparable to those of major depression. Frontal subcortical pathways modulate positive emotions and the reward response [40], and abnormalities in those pathways may therefore be associated with negative emotions.

Structural abnormalities in the hippocampus and amygdala were not related to subsyndromal depression in cognitively normal participants, which was concordant with most previous studies of late-life major depression [15], [16]. Although some studies [10], [41] have supported a decrease in the size of the hippocampus and amygdala in patients with late-life depression, these studies enrolled patients with early-onset depression as well as those with late-onset depression. The volume of the hippocampus and amygdala may be more associated with the duration than with the severity of depression, considering the inverse correlation between the duration of depression and the volume of the hippocampal and parahippocampal area [14]. Steffens et al. (2011) [16] also reported that patients with depression had a greater reduction in hippocampal volume longitudinally than did non-depressed participants. Therefore, the lack of a significant volume difference in the limbic region between patients with their first subsyndromal depressive episode and non-depressed participants could be due to the short duration of illness in the subjects enrolled in this study. Additionally, our results suggest that structural alterations in the prefrontal area precede those in limbic regions such as the hippocampus or amygdala.

The mechanisms of depression and their relationship to neurodegeneration may differ depending on the stage of cognitive impairment. In contrast to findings in cognitively normal elders, subsyndromal depression was not related to significant reduction in prefrontal, limbic, or WML volume in patients with MCI or AD. Moreover, a trend toward increased amygdala volume was observed in patients with depression and MCI. Increased volume of amygdala can be related with the phenomenon that memory score was higher in depressive MCI participants than non-depressive MCI participants [42].

Few studies have explored brain structural changes in a depressed, cognitively impaired geriatric population; these studies reported that late-life depression was not significantly associated with lobar gray or white matter atrophy [43] but, it was significantly associated with hippocampus and left entorhinal cortex [44]. The mechanisms underlying depressive symptoms in cognitively impaired patients are unclear. However, because late-life depression is a complex process originating from a combination of hereditary factors, psychosocial stressors, and brain atrophy [45], the loss of function experienced by cognitively impaired patients may be associated with physical disabilities and changes in friendships and family relationships that could lead to depression. Therefore, it is possible that subsyndromal depression is more related to psychosocial adversity than to brain atrophy [46]. Another explanation for the present findings is that the brain atrophy associated with AD pathology overrides any effect of other factors specifically related to subsyndromal depression. We found that subsyndromal depression independently predicted cognitive impairment, suggesting that if subjects with MCI or AD have depressive symptoms, they may have less brain atrophy at the same cognitive level than do patients with MCI or AD without depressive symptoms. Further longitudinal studies comparing subjects with and without depression are warranted to examine structural changes related to cognitive decline.

Several features of this study differed from previous reports. First, we measured WML volume and regional cortical volumes instead of using an ordinal scoring system. Numerous visual WML rating scales are available, but these subjective scales display ceiling effects and have poor sensitivity [47]. Therefore, volumetric measurements of WML and of cortical areas may yield more accurate results. Additionally, we evaluated the relationships between subsyndromal depression and structural changes in three cognitive groups. Previous studies have explored only cognitively normal participants or patients with dementia, making it impossible to examine the relationships between depression and structural changes according to cognitive status. Our study had several limitations. Our results are from a cross-sectional study; thus, causality between depression and structural changes cannot be confirmed. We did not enroll patients with major depressive disorder or use any depression scale, so we were unable to explore linear trends in the relationship between brain atrophy and depression severity. The statistical analysis was challenging due to the relatively small sample size, particularly in the subsyndromal depressive group. Therefore, the findings of this study should be interpreted preliminary and, in order to find this result to be generalized, it should be replicated.

Conclusively, we found frontal lobe abnormalities in subsyndromal depressive patients, which showed same abnormalities in major depressive patients in previous studies. We also found that the effects of subsyndromal depression on brain structure did not differ significantly according to the level of cognitive impairment. The results suggest that brain atrophy due to cognitive impairment may outweigh brain changes associated with late-life subsyndromal depression.
